# Microtubule and Actin Interplay Drive Intracellular c-Src Trafficking

**DOI:** 10.1371/journal.pone.0148996

**Published:** 2016-02-11

**Authors:** Christopher Arnette, Keyada Frye, Irina Kaverina

**Affiliations:** Department of Cell and Developmental Biology, Vanderbilt University Medical Center, Nashville, Tennessee, United States of America; University of Birmingham, UNITED KINGDOM

## Abstract

The proto-oncogene c-Src is involved in a variety of signaling processes. Therefore, c-Src spatiotemporal localization is critical for interaction with downstream targets. However, the mechanisms regulating this localization have remained elusive. Previous studies have shown that c-Src trafficking is a microtubule-dependent process that facilitates c-Src turnover in neuronal growth cones. As such, microtubule depolymerization lead to the inhibition of c-Src recycling. Alternatively, c-Src trafficking was also shown to be regulated by RhoB-dependent actin polymerization. Our results show that c-Src vesicles primarily exhibit microtubule-dependent trafficking; however, microtubule depolymerization does not inhibit vesicle movement. Instead, vesicular movement becomes both faster and less directional. This movement was associated with actin polymerization directly at c-Src vesicle membranes. Interestingly, it has been shown previously that c-Src delivery is an actin polymerization-dependent process that relies on small GTPase RhoB at c-Src vesicles. In agreement with this finding, microtubule depolymerization induced significant activation of RhoB, together with actin comet tail formation. These effects occurred downstream of GTP-exchange factor, GEF-H1, which was released from depolymerizing MTs. Accordingly, GEF-H1 activity was necessary for actin comet tail formation at the Src vesicles. Our results indicate that regulation of c-Src trafficking requires both microtubules and actin polymerization, and that GEF-H1 coordinates c-Src trafficking, acting as a molecular switch between these two mechanisms.

## Introduction

c-Src is a non-receptor tyrosine kinase that has been implicated in pathways regulating angiogenesis, invasion and metastasis, cell migration, endocytosis, and many others [1–6]. Activation of c-Src is associated with its translocation to the plasma membrane where c-Src interacts with a number of important effectors [7]. Therefore, the subcellular localization of c-Src is critical to its function.

Studies have indicated that inactive c-Src can be localized to the perinuclear region, colocalizing with endosomal and Golgi markers [8], and upon activation, it can be subsequently targeted to the cell periphery [9]. However, at present, the mechanism by which c-Src is localized in response to cellular cues is still quite not understood. In neuronal growth cones, trafficking of endosomal c-Src was identified as a MT-dependent process that allowed for bidirectional trafficking along the MT network [10, 11]. As such, microtubule depolymerization lead to the inability of c-Src to properly recycle from the neuronal growth cone. Additionally, it has been shown that c-Src delivery to the plasma membrane is an actin polymerization-dependent process that relies on small GTPase RhoB and actin nucleating machinery components (Scar1/WAVE1 and mDia2), found at the membrane of c-Src-associated endosomes. [12, 13]. This, existing data suggest that both MTs and actin are important for c-Src positioning; however, it is not clear whether and how these mechanisms cooperate within a cell.

Here, we applied high-resolution confocal microscopy and biochemical techniques to determine the mechanism of c-Src trafficking, as well as the activation status of the molecular players involved. We determined a mechanism whereby MTs and actin combine their efforts for efficient trafficking of c-Src-associated endosomes; moreover, we provide evidence of tight coordination between these mechanisms, whereby GEF-H1-dependent RhoB activation serves as a switch.

## Materials and Methods

### Cells

Immortalized human retinal pigment epithelial cells, hTert-RPE1 (Clontech), were maintained in DMEM/F12 with 10% fetal bovine serum (FBS) and 5% L-glutamine. Rat aortic vascular smooth muscle cells (A7r5) were grown in DMEM with 10% fetal calf serum and 5% L-glutamine. Cells were plated on fibronectin-coated glass coverslips 24 hours before experiments. In all live cell experiments, cells were maintained on the microscope stage at 37°C under mineral oil for media equilibrium maintenance.

### Treatments

For MT depolymerization, cells were incubated in 2.5μg/ml nocodazole (Sigma, St. Louis, MO) 2 hours prior to imaging. For RBD-RhoB pulldowns, cells were incubated in nocodazole for 30 minutes prior to cell lysis and incubation.

### Expression Constructs

GFP-Src has previously been described [12] was provided by Dr. Giulio Superti-Furga (EMBL, Heidelberg, Germany). The c-Src and linker region coding sequence was excised from GFP-Src by digestion with BglII/AgeI and cloned into BglII/AgeI pmCherry-N1 to create pm-cherry-Src. GFP-GEF-H1 and GFP-GEF-H1 1–573 (gifts from Mira Krendel, Syracuse, NY). GEF-DN and GFP-RhoB were purchased from addgene (Cambridge, MA). 3x-mCherry-EMTB (gift from William Bement, Madison, WI) was used for MT visualization. RFP-cortactin was provided by Marko Kaksonen (U.C. Berkeley) and used to visualize actin comet tail formation. RPE1 and A7r5 cells were transfected with Fugene6 (Roche, Indianapolis, IN) according to manufacturer's protocols.

### Antibodies and Immunofluorescence Details

For actin comet tail identification, mouse monoclonal antibody against cortactin (1:1000; Upstate, Lake Placid, NY) was used. Cells were fixed (5' at 20°.) in 100% methanol. Alexa488-conjugated highly cross-absorbed goat anti-mouse IgG antibody (1:500; Molecular Probes, Invitrogen, Eugene, OR) was used as secondary antibodies.

### Confocal and live cell imaging

Leica TCS SP5 confocal laser scanning microscope with an HCX PL APO 100x oil lens NA 1.47 was used for taking confocal stacks of fixed cells.

Live cells plated on MatTek glass bottom dishes were maintained at 37°C by heated stage (Warner Instruments). Single plane confocal video sequences were taken were taken by Yokogawa QLC-100/CSU-10 spinning-disk head (Visitec assembled by Vashaw) attached to Nikon TE2000E microscope using CFI PLAN APO VC 100X OIL lens, NA 1.4 with 1.5× intermediate magnification and back-illuminated EM-CCD camera Cascade 512B (Photometrics) driven by IPLab software (Scanalytics). 75 mW 488/568 Krypton-Argon laser (Melles Griot) with AOTF was used for 2-color excitation. Custom double dichroic mirror and filters (Chroma) in a filter wheel (Ludl) were used in the emission light path.

### Western blot analysis

Western blotting was performed with the Protein Electrophoresis and Western Blotting System (Bio-Rad, Hercules, CA). For western blotting, a rabbit polyclonal antibody against RhoB (1:500; Cell Biolabs, San Diego, CA) was used. Nitrocellulose membrane was incubated with primary and then secondary antibody (LI-COR Bioscience, IRDye™ 800, Lincoln, NE) diluted in Odyssey Blocking Buffer with 0.2% Tween-20 to lower background. Odyssey Infrared Imaging System (LI-COR Bioscience, Lincoln, NE) was used for membrane scanning.

### RhoB Activation

Active RhoB levels were determined as in [14, 15]. Cells were stimulated for 30 minutes, washed with ice-cold PBS and lysed in in buffer containing 125mM Hepes, pH 7.5, 750 mM NaCL, 5% NP-40, 50mM MgCl_2_, 5mM EDTA, and 10% glycerol, supplemented with protease inhibitors. Lysates were cleared by centrifugation (14,000 rpm, 10min). A sample of supernatant was removed and lysates incubated with RBD beads (Cell BioLabs) for 1 hour at 4°C. Beads were washed three times with lysis buffer and protein cleaved by addition of 2x sample buffer. Samples were boiled and analyzed by Western blotting. Levels of activated RhoB normalized to total Rho for each experiment according to the manufacturer manual [(active RhoB/Total RhoB) x 100], and the average across a set of experiment was taken.

### c-Src vesicle trafficking analysis

To analyze vesicle directionality and velocity in control cells and nocodazole-treated cells, 5 min one-channel confocal sequences (5 sec/frame) of cells expressing GFP-Src were used. Individual vesicles were manually tracked using the MTrackJ plugin of ImageJ by following movements of the vesicles as visualized by GFP-Src expression. 50 vesicles per condition (control or nocodazole-treated) were analyzed. Average velocity was calculated from instantaneous velocity measurements obtained from the MTrackJ plugin of ImageJ. Directional persistence of vesicles was quantified as final distance of vesicle relocation divided by total vesicle track length.

### Cortactin quantification

Leica confocal z-sections were obtained of control and nocodazole-treated cells stained for cortactin. Maximum intensity z-projections of three central slices were used for thresholding of cortactin puncta. Number of cortactin puncta under each condition was automatically quantified by ImageJ analysis. Similar methods were used upon expression of various GEF-H1 constructs.

### Cortactin association with c-Src vesicles

Cortactin association analysis of control and nocodazole-treated cells was restricted to 4μm^2^ ROIs. Cortactin association was scored 2.5 minutes after initiation of image acquisition. Number of cortactin-associated c-Src vesicles was visually scored and divided by the total number of c-Src vesicles within an ROI to determine association percentage.

### Mander’s Coefficient Distribution

To determine the degree of overlap, the first frame of live-cell confocal imaging sequences in A7r5 cells was used. Images were subjected to thresholding in the JaCOP plugin of ImageJ and Mander’s coefficient was automatically calculated for the degree of overlap of GFP-RhoB and mCherry-Src.

### Statistical Analysis

Statistical significance was determined by Students t-test (two-tailed, unpaired). A p-value of < .05 was considered statistically significant. Asterisks indicate statistical significance.

## Results and Discussion

### c-Src traffics bidirectionally on MTs

**Fig 1:**
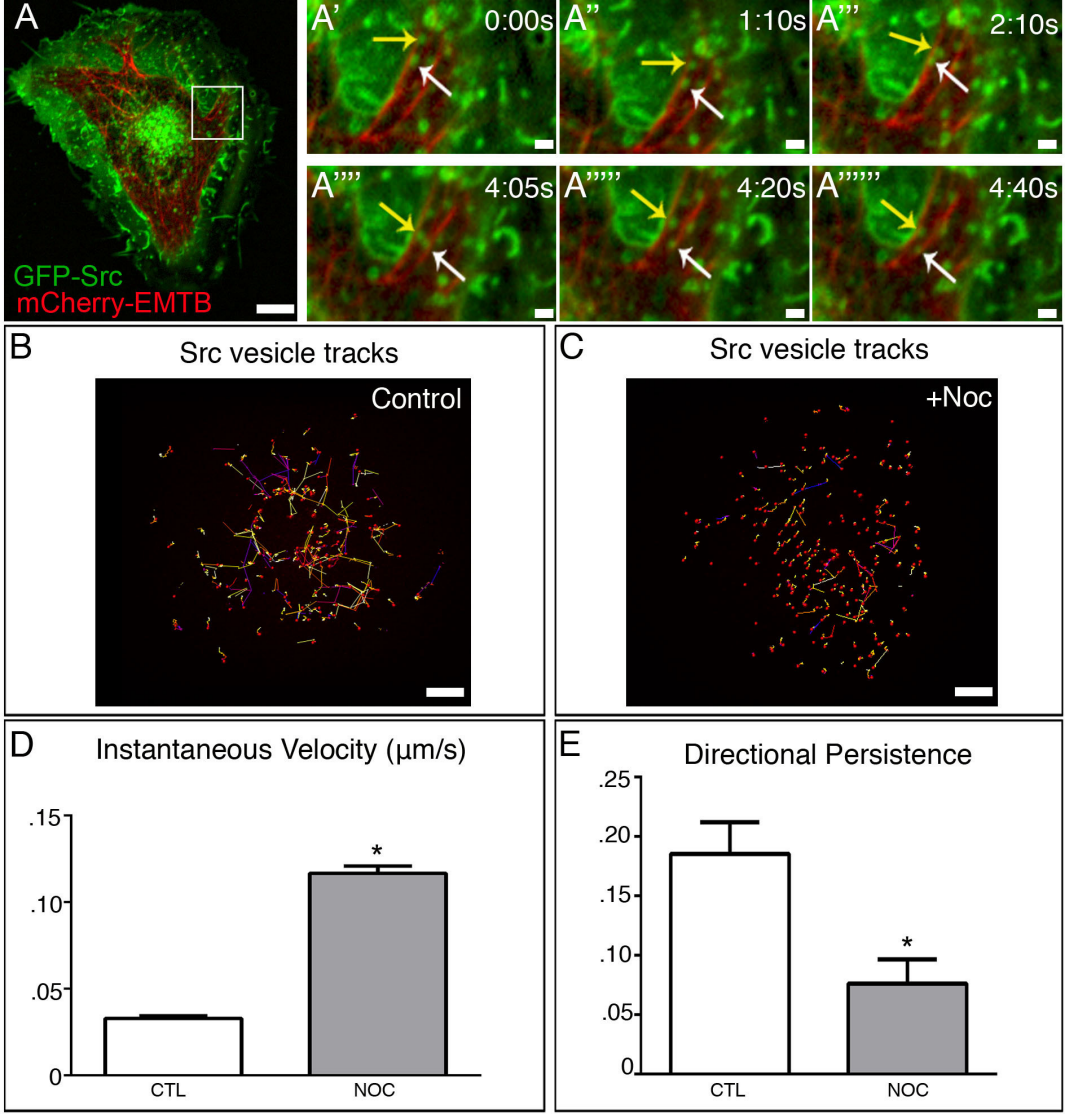
c-Src exhibits bimodal trafficking. (A) Detection of GFP-Src (green) and 3x-mCherry-EMTB (red) in time-lapse confocal movie of an A7r5 cell. Area in box is enlarged to the right. Bar, 5μm. (A’-A”””) Imaging sequence illustrates that GFP-Src is trafficked bidirectionally along MTs in *A*. Bar, 2μm (B) GFP-Src vesicles exhibit long, directional movement in time-lapse confocal movie of an A7r5 control (5s/frames). Representative tracks. Bar, 5μm. (C) GFP-Src vesicles exhibit short, randomized movement in time-lapse confocal movie of an A7r5 control (5s/frames). Representative tracks. Bar 5μm. (D) GFP-Src vesicles exhibit increased velocity in response to MT depolymerization. (E) GFP-Src vesicles show reduced directional persistence following nocodazole treatment. N = 5 cells/condition, 50 vesicles/cell. See also S1 Movie. Error bars present SEM. Asterisks indicate p-value < .05.

The goal of this study was to investigate the molecular mechanism by which c-Src is trafficked and whether this trafficking required the cooperative action of both the MT and actin cytoskeleton. To address this, we utilized a cell culture system including Retinal Pigment Epithelial cells (RPE1) and rat aortic vascular smooth muscle cells (VSMCs). Because previous reports have shown that c-Src can be trafficked independently by MTs (MTs) [10], we first determined whether this mechanism existed in our system. We found that GFP-Src on endosomal membranes was trafficked bidirectionally along MTs (Fig 1A–1A’’’’’’, and S1 Fig; see also S1 Movie) from the cell center and cell periphery both in A7r5 and RPE cells. Interestingly, depolymerization of MTs by the MT-depolymerizing drug, nocodazole, did not inhibit GFP-Src movement, but instead disrupted the trajectory of GFP-Src vesicles, restricting them spatially (Fig 1B and Fig 1C). Analysis of GFP-Src vesicle velocity under control and nocodazole-treated conditions revealed that upon MT depolymerization, GFP-Src vesicle velocity significantly increased (Fig 1D and S1 Fig) in both cell types. Additionally, analysis of vesicle directionality under these conditions revealed that vesicle movement becomes highly randomized as compared to control (Fig 1E and S1 Fig). The changes observed in both RPE and A7r5 suggest a more global, rather than a cell-type specific mechanism of c-Src trafficking. We hypothesize that this bimodal mechanism of c-Src trafficking may represent two distinct mechanisms that have converged to increase the efficiency of c-Src vesicle trafficking for both long and short delivery.

### MT depolymerization induces actin comet tail formation through regulating GEF-H1

Previous reports have shown that c-Src vesicles are associated with actin nucleating machinery, particularly small GTPase RhoB, adaptor protein Scar1/Wave1 and actin filament regulatory protein, mDia2 [12]. RhoB activation facilitates formation of polymerizing actin clouds around these vesicles, which was suggested to participate in transportation of c-Src to its sites of activity. However, the exact role of RhoB-dependent actin polymerization in c-Src vesicle movement (whether c-Src is trafficked along or pushed by polymerizing actin) is unclear. The regulation of this process in the cellular context is also elusive. We hypothesized that non-directional movement of c-Src vesicles, which we observed stimulated upon MT depolymerization, is an actin-dependent process.

**Fig 2:**
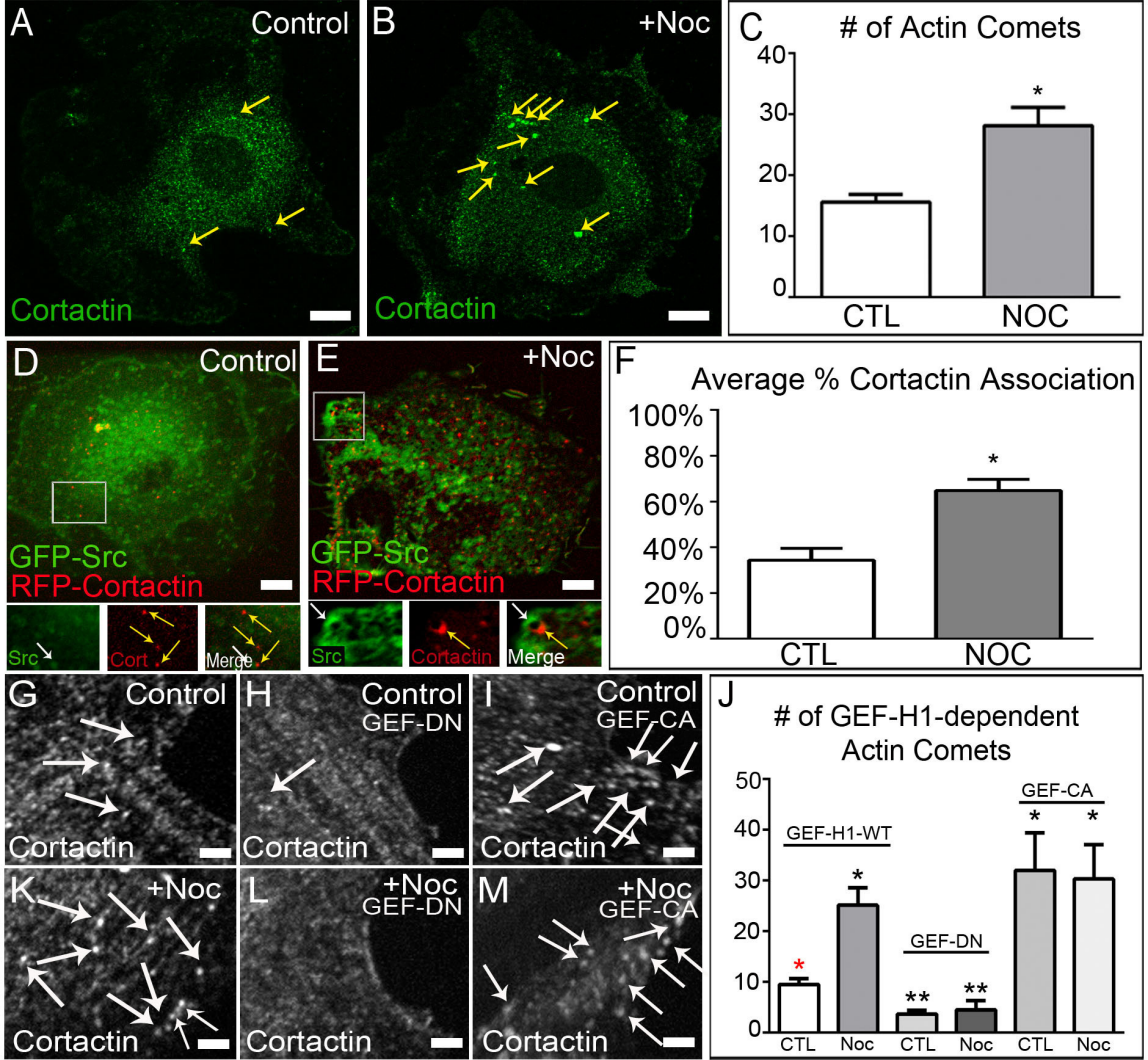
GEF-H1 regulates formation of actin comet tails at c-Src vesicle membranes. (A-B) Control and nocodazole-treated A7r5 cells fixed and stained for cortactin (green). Immunostaining. Bar, 5μm. (C) Quantification of actin comet tail formation in control and nocodazole-treated cells reveals a two-fold increase upon MT depolymerization. Representative examples out of 30 cells/condition. Error bars indicate s.e.m. (D-E) Live-cell imaging of control and nocodazole-treated A7r5 cells expressing GFP-Src and RFP-Cortactin. Area in box is enlarged below. Bar 5μm. See also S3 Movie. (F) Quantification reveals that MT depolymerization induces increased actin comet formation at c-Src vesicle membranes. (G and K) Z-section of A7r5 cells expressing wild-type GEF-H1 pre- and post-nocodazole fixed and immunostained for cortactin. Images illustrate an increase in cortactin puncta (white arrows) following MT depolymerization Bar, 5μm. (H and L) Z-section of A7r5 cells expressing dominant-negative GEF-H1 pre- and post-nocodazole demonstrates the role of GEF-H1 in forming actin comets. Bar, 5μm. (I and M) Z-section of A7r5 cells expressing constitutively active GEF-H1 pre- and post-nocodazole show high number of actin comets in the presence or absence of MTs. Bar, 5μm. (J) Quantification of actin comet tail formation after expression of various GEF-H1 constructs. Representative examples out of 30 cells/condition. Error bars indicate SEM. Asterisks indicate p-value < .05.

To determine the role of actin polymerization in c-Src vesicular movement upon MT depolymerization, we applied immunofluorescence techniques to provide an overall idea of the magnitude of actin polymerization occurring. We found that upon MT depolymerization, the number of cortactin puncta (concentrated foci of actin polymerization), formed directly at c-Src vesicle membranes, significantly increased (Fig 2A–2C and S3 Fig). As Scar1/Wave1 has been implicated in as important for mediating actin polymerization at c-Src vesicles, we examined its role in our system by utilizing an inhibitor of Arp2/3, CK-666. Since the effects of Scar1/Wave1 on the actin cytoskeleton are mediated through interaction with the Arp2/3 complex, we hypothesized that inhibiting Arp2/3 activity would abolish actin comet tail formation following nocodazole treatment. We found that treatment with CK-666 following nocodazole treatment blocked c-Src trafficking (See S2 Movie). Additionally, we applied live-cell confocal microscopy imaging of cells co-expressing GFP-Src and RFP-cortactin, as a marker of polymerizing actin. Under these conditions, percentage of cortactin puncta associated with c-Src vesicles was also increased upon MT depolymerization (Fig 2D–2F; see also S3 Movie). Localization of cortactin puncta of the rear side of moving vesicles suggested that actin polymerizing at c-Src vesicles in the shape of “actin comet tails” directly facilitates their transport, rather than myosin motors.

Furthermore, these data also suggest that MT regulation of some factor is perturbed upon MT depolymerization, allowing said factor to interact with actin nucleating machinery. As GEF-H1, a Rho GEF regulated by MT binding [16], has been implicated in being a mediator of cytoskeletal crosstalk [17, 18], we examined whether it was involved in potentiating the formation of actin comet tails upon MT depolymerization. Utilizing cortactin as a marker of actin comet tails, we tested their formation by immunostaining of VSMCs and RPE cells transiently expressing various GFP-GEF-H1 constructs. GFP-GEF-H1-wild type (GEF-WT) expressing cells exhibited few actin comet tails prior to MT depolymerization, but this significantly increased after treatment with nocodazole (Fig 2G, 2K and 2J; See also S3 Fig). In cells expressing GFP-GEF-H1-dominant negative (GEF-DN), actin comet tail formation was inhibited both in control and nocodazole treated cells (Fig 2H, 2L and 2J; See also S3 Fig), further implicating GEF-H1 in mediating cytoskeletal crosstalk in our cells. Furthermore, cells expressing GFP-GEF-H1-constitutively active (GEF-CA), which is unable to bind MTs, exhibited increased actin comet tail formation in the presence and absence of MTs, similar to levels seen in nocodazole-treated control (Fig 2I, 2M and 2J; See also S3 Fig). This data places GEF-H1 within the molecular pathway and delegates a role for GEF-H1 in regulating this crosstalk. We propose that actin comet tail formation directly at c-Src vesicle membranes, mediated through the action of GEF-H1, might serve as a secondary mechanism to increase the efficiency of c-Src trafficking.

### RhoB associates with c-Src vesicle membranes

**Fig 3:**
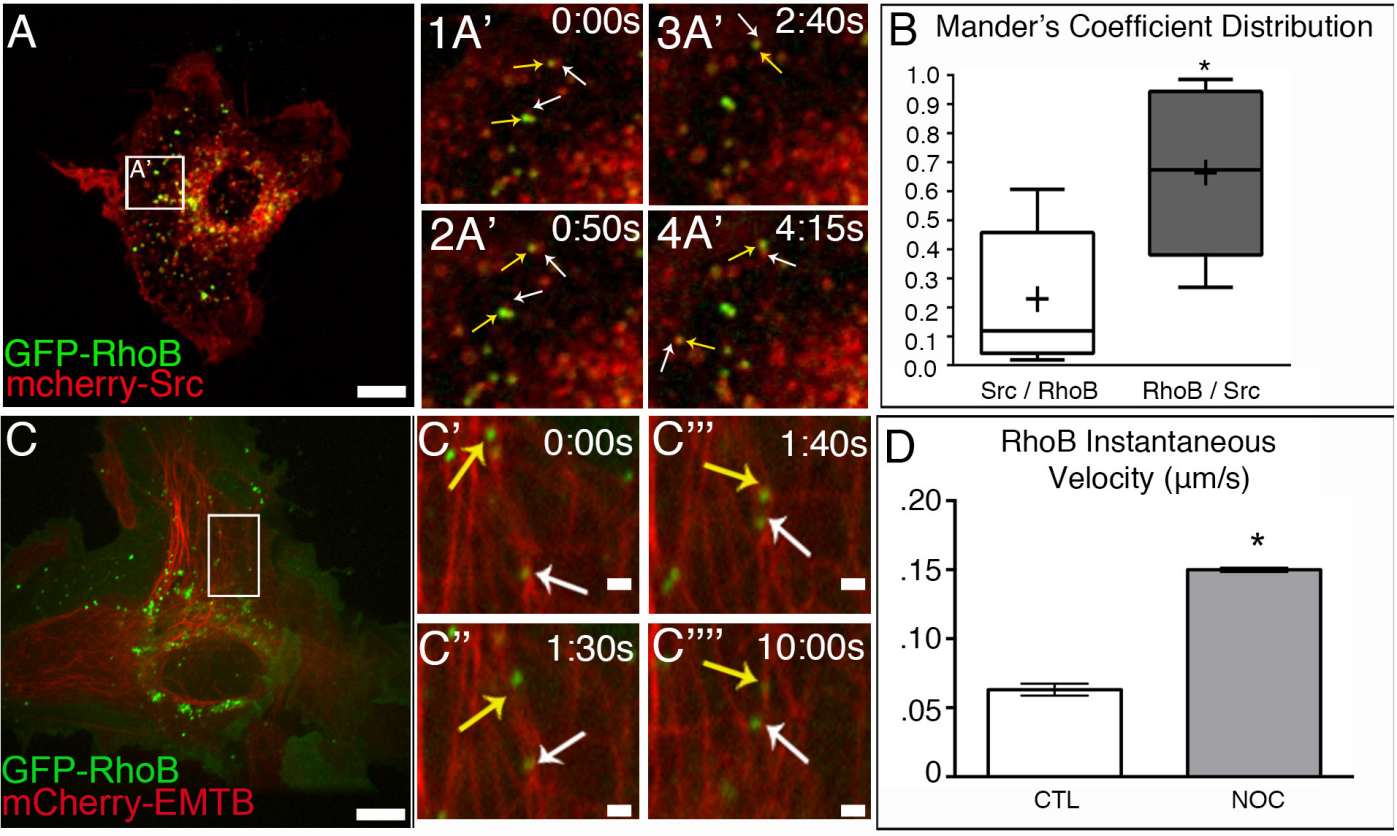
RhoB associates with c-Src vesicles is trafficked along MTs. (A) A7r5 cell expressing GFP-RhoB (green) and mCherry-Src (red). Area in box is enlarged to the right. Spinning disk confocal. Bar, 5μm. (A’1 and A’2) Time-lapse confocal microscopy shows that GFP-RhoB (yellow arrows) can associate with a subset of mcherry-Src vesicles (white arrows. See also S4 Movie. (B) Mander’s coefficient distribution reveals that the majority of GFP-RhoB vesicles are associated with mCherry-Src. N = 10 cells. (C) A7r5 cells expressing GFP-RhoB (green) and 3X-mCherry-EMTB (red). Area in box is enlarged to the right. Spinning disk confocal. Bar, 5μm. (C’–C””) Time-lapse confocal microscopy reveals vesicles GFP-RhoB vesicles move along MTs (yellow and white arrows). See also S5 Movie. (D) GFP-RhoB vesicles exhibit increased velocity in response to MT depolymerization. N = 5 cells/condition, 50 vesicles/cell. Bar, 2μm. Error bars represent SEM. Asterisks indicate p-value < .05.

It has previously been identified that actin-dependent trafficking of c-Src vesicles relied on RhoB localization at the vesicle membrane [12]. To confirm whether c-Src and RhoB localized within the same vesicle, cells expressing GFP-RhoB and mCherry-Src were imaged by spinning-disk confocal microscopy. Our data shows that all GFP-RhoB puncta colocalized with a subset of mCherry-Src vesicles (Fig 3A–3A’2 and Fig 3B; see also S4 Movie), particularly at one side of the vesicle membrane. Positioning of RhoB at c-Src vesicles membranes may serve as a mechanism by which actin polymerization can be asymmetrically localized at c-Src vesicles to support asymmetric pushing force distribution and fast vesicle movement, as expression of dominant-negative RhoB inhibited vesicle movement [12].

Furthermore, to test that MTs are capable of transportation of the same endosome population, we tested whether RhoB-associated vesicles could also be transported along MTs. We utilized live-cell confocal imaging of cells expressing GFP-RhoB and 3x-mCherry-EMTB and tracked vesicle movement. Our data shows that GFP-RhoB vesicles could traffic bidirectionally along MTs (Fig 3C–3C’’’’; see also S5 Movie) in a manner similar to c-Src vesicles. Analysis of GFP-RhoB velocity and directionality indicated that GFP-RhoB vesicles exhibited similar increases in velocity following MT depolymerization (Fig 3D), further suggesting that both c-Src and RhoB are on the same vesicles. These data indicate that both MT and actin-dependent mechanisms can potentially move RhoB-containing c-Src-associated endosomes.

It is noteworthy that MT depolymerization also led to increased activation of c-Src at the endosomal membrane (S2 Fig), which might contribute to activation of actin polymerization machinery; however, no asymmetry was observed in localization of phosphor-Src at the endosome membrane, indicating that local actin nucleation sites and actin comet tail formation is not guided by c-Src activation

### RhoB is activated by MT depolymerization

**Fig 4:**
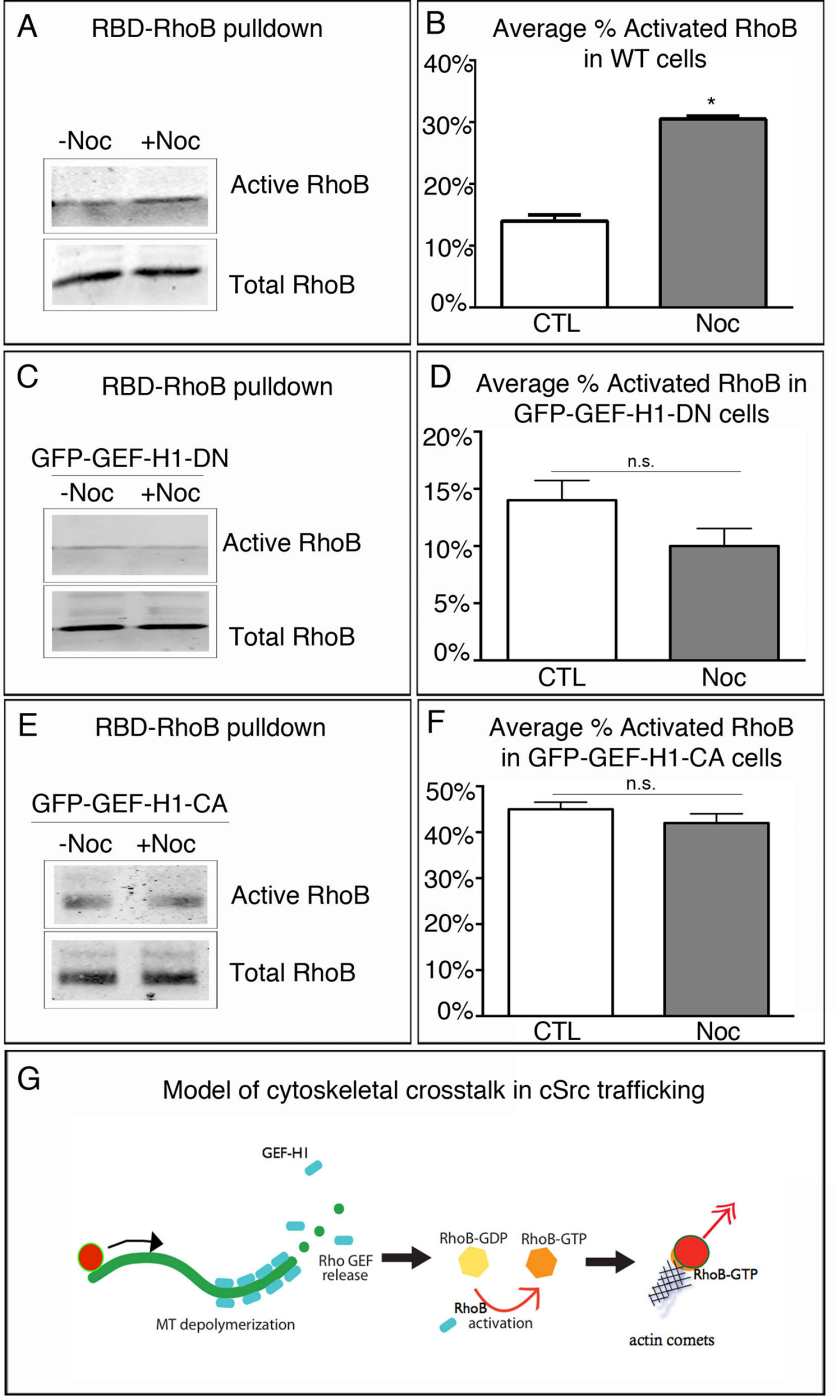
RhoB is activated by MT depolymerization. (A) Western blot analysis of RhoB activation in response to MT depolymerization in control and nocodazole-treated RPE1 cells. (B) Quantification of average percent of RhoB activation from A reveals that RhoB activation is significantly higher following MT depolymerization. (C) Western blot analysis of RhoB activation in GFP-GEF-H1-DN expressing cells pre- and post-nocodazole treatment. (D) Quantification of the average percent of activation under both conditions reveals that inhibition of GEF-H1 activity significantly impairs RhoB activation. (E) Western blot analysis of RhoB activation in GFP-GEF-H1 constitutively active expressing cells. (F) Quantification reveals that constitutive activation of GEF-H1 leads to high levels of RhoB even in the presence of MTs. Pulldowns repeated in triplicate. Error bars indicate SEM. Asterisks indicate p-value < .05. (G) Model of cytoskeletal crosstalk for c-Src delivery.

It has been shown that GEF-H1 can function as a RhoB GEF to promote RhoB activation [19, 20]. We hypothesized that GEF-H1 facilitates the formation of actin comet tails via activation of RhoB at the endosomal membrane. To assess RhoB activation in the absence or presence of MT depolymerization, RBD-RhoB pulldowns were performed and immunoblotted for total and activated RhoB. As A7r5 cells divide slowly, they do not lend themselves to biochemical studies. Thus, to test RhoB activation, RPE cells, which are a robust source of cells and protein, were used. Analysis revealed that upon MT depolymerization RhoB activity doubled as compared to control conditions (Fig 4A and Fig 4B). Thus, RhoB activity can be modulated by the presence or absence of an intact MT network. To test the effect of GEF-H1 activity on RhoB activation, we expressed a GEF-DN mutant to abolish GEF-H1 activity, and then performed RBD-RhoB pulldowns in the absence and presence of nocodazole. Under these conditions, basal levels of activated RhoB were similar to those seen in untreated wild-type cells; however, treatment with nocodazole did not significantly enhance RhoB activation levels in these cells (Fig 4C and Fig 4D). This inability to modulate RhoB levels in these cells further implicates that GEF-H1 is required for the observed RhoB activation. It also suggests a lack of redundant regulation of RhoB by other GEFs, as RhoB activation levels were significantly diminished to basal levels after GEF-DN expression. We further analyzed how expression of constitutively active GFP-GEF-H1 influenced activation of RhoB under similar conditions. In the absence of nocodazole treatment, GEF-CA cells had significantly higher levels of activation as compared to control and GEF-DN expressing cells (compare 45% ± .02 to 14% ± .02 and 14% ± .02). Moreover, MT depolymerization by nocodazole did not significantly change activated RhoB levels in GEF-CA expressing cells (Fig 4E and Fig 4F), while levels were still significantly higher than in treated control and GEF-DN expressing cells (compare 42% ± .02 to 28% ± .02 and 10% ± .01). Therefore, we hypothesize that this activation of RhoB is mediated by GEF-H1 release from depolymerizing MTs.

This result explains our observation that inhibition of GEF-H1 activity leads to loss of actin comet tail formation (Fig 2). This evidence also places GEF-H1 within the molecular pathway that defines the mechanism of c-Src trafficking and positioning (Fig 4G), further providing a complete molecular mechanism that defines targets for disease therapies. We hypothesize that MTs provide long-range, directional trafficking through the cell body, while actin comet tails are predominantly formed at the cell periphery, promoted by MT depolymerization events as a part of dynamic instability. In this scenario, dynamic MTs could fine-tune the final step in delivery of c-Src vesicle to the plasma membrane through localized release of GEF-H1 for RhoB activation. Actin polymerization directly at c-Src vesicle membranes could provide the necessary forces essential for bringing c-Src within close proximity to the plasma membrane and efficient membrane fusion. In this way, the microtubule and actin cytoskeletons act as conduits for the efficient delivery of c-Src, localizing components, such as GEF-H1, that facilitate this bimodal transport, although not directly interacting with c-Src. This mechanism probably contributes to the proper spatial and temporal regulation of c-Src necessary to prevent aberrant signaling.

## Supporting Information

S1 Fig c-Src exhibits bimodal trafficking, related to Fig 1. (A) Detection of GFP-Src (green) and 3x-mCherry-EMTB (red) in time-lapse confocal movie of an RPE cell. Bar, 5μm. Area in box is enlarged to the right. Bar, 2μm. N = 5 cells/condition, 50 vesicles/cell.
(EPS)

S2 Fig MT depolymerization increases activation of c-Src, related to Fig 1. Analysis of fluorescence intensity in untreated versus nocodazole-treated cells reveals a significant increase in phosphorylated Src staining following MT depolymerization. Error bars indicate SEM. Bar, 5μm. (B) GFP-Src vesicles exhibit increased velocity in response to MT depolymerization. (C) GFP-Src vesicles show reduced directional persistence following nocodazole treatment. N = 5 cells/condition, 50 vesicles/cell. Error bars present SEM. Asterisks indicate p-value < .05.
(EPS)

S3 Fig GEF-H1 regulates formation of actin comet tails at c-Src vesicle membranes, related to Fig 2. (A-B) Control and nocodazole-treated RPE cells fixed and stained for cortactin (green). Immunostaining. Bar, 5μm. (C) Quantification of actin comet tail formation in control and nocodazole-treated cells reveals a two-fold increase upon MT depolymerization. Representative examples out of 10 cells/condition. Error bars indicate SEM. (G and K) Z-section of RPE cells expressing wild-type GEF-H1 pre- and post-nocodazole fixed and immunostained for cortactin. Images illustrate an increase in cortactin puncta (white arrows) following MT depolymerization Bar, 5μm. (H and L) Z-section of RPE cells expressing dominant-negative GEF-H1 pre- and post-nocodazole demonstrates the role of GEF-H1 in forming actin comets. Bar, 5μm. (I and M) Z-section of RPE cells expressing constitutively active GEF-H1 pre- and post-nocodazole show high number of actin comets in the presence or absence of MTs. Bar, 5μm. (J) Quantification of actin comet tail formation after expression of various GEF-H1 constructs. Representative examples out of 15 cells/condition. Error bars indicate SEM. Asterisks indicate p-value < .05.
(EPS)

S1 Movie c-Src moves bidirectionally along MTs, related to Fig 1. A7r5 cells transfected with GFP-Src (green) and 3X-cherry-EMTB (red). Images were analyzed by time-lapse spinning disk confocal microscopy (Nikon TE2000E, Nikon, Inc.). Single confocal plane. Frames were taken every five seconds for 5 minutes. Cropped region (see Fig 1A–1A’’’’). White and yellow arrows highlight c-Src vesicle movement.
(MOV)

S2 Movie Inhibiting Scar1/Wave1 activity inhibits vesicle movement following nocodazole addition, related to Fig 2. A7r5 cells transfected with GFP-Src and treated with 2.5ug/ml nocodazole for 2 hours followed by addition of 50μM ck666 inhibitor. Images were analyzed by time-lapse spinning disk confocal microscopy (Nikon TE2000E, Nikon, Inc). Single confocal plane. Frames were taken every five seconds for 5 minutes.
(MP4)

S3 Movie MT depolymerization induces actin comet formation, related to Fig 2. A7r5 cells transfected with GFP-Src (green) and RFP-Cortactin (red) and treated with 2.5ug/ml nocodazole for two hours. Images were analyzed by time-lapse spinning disk confocal microscopy (Nikon TE2000E, Nikon, Inc). Single confocal plane. Frames were taken every five seconds for 5 minutes. Cropped region (see Fig 2E). White and yellow arrows highlight actin polymerization.
(MOV)

S4 Movie RhoB associates with c-Src vesicles, related to Fig 3. A7r5 cells expressing GFP-RhoB (green) and mCherry-Src (red). Images were analyzed by time-lapse confocal microscopy (Nikon, TE2000E, Nikon, Inc.) Single confocal plane. Cropped region (see Fig 3A–3A’2). White and yellow arrows highlight c-Src and RhoB association.
(MOV)

S5 Movie RhoB vesicles are trafficked along MTs, related to Fig 3. A7r5 cells transfected with GFP-RhoB (green) and 3X-GFP-EMTB (green). Images were analyzed by time-lapse confocal microscopy (Nikon, TE2000E, Nikon, Inc.) Single confocal plane. Cropped region (see Fig 3C–3C’’’’). White arrow highlights RhoB vesicle movement.
(MOV)
